# ^1^H/^31^P Polarization Transfer at 9.4 Tesla for Improved Specificity of Detecting Phosphomonoesters and Phosphodiesters in Breast Tumor Models

**DOI:** 10.1371/journal.pone.0102256

**Published:** 2014-07-18

**Authors:** Jannie P. Wijnen, Lu Jiang, Tiffany R. Greenwood, Wybe J. M. van der Kemp, Dennis W. J. Klomp, Kristine Glunde

**Affiliations:** 1 Johns Hopkins University In vivo Cellular and Molecular Imaging Center, The Russell H. Morgan Department of Radiology and Radiological Science, Division of Cancer Imaging Research, The Johns Hopkins University School of Medicine, Baltimore, Maryland, United States of America; 2 Department of Radiology, University Medical Centre Utrecht, Utrecht, Netherlands; 3 Sidney Kimmel Comprehensive Cancer, Johns Hopkins University School of Medicine, Baltimore, Maryland, United States of America; National Research Council of Italy, Italy

## Abstract

**Purpose:**

To assess the ability of a polarization transfer (PT) magnetic resonance spectroscopy (MRS) technique to improve the detection of the individual phospholipid metabolites phosphocholine (PC), phosphoethanolamine (PE), glycerophosphocholine (GPC), and glycerophosphoethanolamine (GPE) *in vivo* in breast tumor xenografts.

**Materials and Methods:**

The adiabatic version of refocused insensitive nuclei enhanced by polarization transfer (BINEPT) MRS was tested at 9.4 Tesla in phantoms and animal models. BINEPT and pulse-acquire (PA) ^31^P MRS was acquired consecutively from the same orthotopic MCF-7 (n = 10) and MDA-MB-231 (n = 10) breast tumor xenografts. After *in vivo* MRS measurements, animals were euthanized, tumors were extracted and high resolution (HR)-MRS was performed. Signal to noise ratios (SNRs) and metabolite ratios were compared for BINEPT and PA MRS, and were also measured and compared with that from HR-MRS.

**Results:**

BINEPT exclusively detected metabolites with ^1^H-^31^P coupling such as PC, PE, GPC, and GPE, thereby creating a significantly improved, flat baseline because overlapping resonances from immobile and partly mobile phospholipids were removed without loss of sensitivity. GPE and GPC were more accurately detected by BINEPT *in vivo*, which enabled a reliable quantification of metabolite ratios such as PE/GPE and PC/GPC, which are important markers of tumor aggressiveness and treatment response.

**Conclusion:**

BINEPT is advantageous over PA for detecting and quantifying the individual phospholipid metabolites PC, PE, GPC, and GPE *in vivo* at high magnetic field strength. As BINEPT can be used clinically, alterations in these phospholipid metabolites can be assessed *in vivo* for cancer diagnosis and treatment monitoring.

## Introduction

Elevated concentrations of water-soluble phospholipid metabolites such as the phosphomonoesters (PMEs) phosphocholine (PC) and phosphoethanolamine (PE) and the phosphodiesters (PDEs) glycerophosphocholine (GPC) and glycerophosphoethanolamine (GPE) are a metabolic hallmark of cancer [1], [2]. ^1^H and ^31^P magnetic resonance spectroscopy (MRS) are able to detect this activated phospholipid metabolism in cancers *in vivo*, which can be used clinically for cancer diagnosis and treatment monitoring [2]. However, the total choline (tCho) signal detected by ^1^H MRS cannot be spectrally resolved into individual metabolites, mainly free choline (Cho), PC and GPC, due to the low spectral resolution *in vivo* at clinical field strengths of 1.5 and 3 Tesla (T), nor can it be resolved at high field strengths of 4 and 7 T [3], [4], [5]. Non-invasive detection of these individual phospholipid metabolites is of high interest as it has been shown that, for example, the PC/GPC ratio can increase with increasing aggressiveness of breast cancer cells [6]. With ^31^P MRS, individual PME and PDE signals can be detected, however, it suffers from relatively low signal to noise ratio (SNR) due to the intrinsically low sensitivity of the ^31^P nucleus [7], [8]. Even at high field strength, the detection of individual PE, PC, GPE and GPC is difficult, particularly in heterogeneous cancer tissues in which the homogeneity of the magnetic field is poor [9]. Another significant problem for quantitative *in vivo* detection with ^31^P MRS is that these signals typically overlap with signals of other molecules with ^31^P nuclei, such as sugar phosphates and immobile membrane phospholipids (e.g. phosphatidylcholine, phosphatidylethanolamine), which cause a broad, uneven baseline, which in turn severely hampers the accuracy of metabolite measurement and quantification [10]. Accurate measurement and quantification of these phospholipid metabolites is particularly important in the clinic as consistent changes in phospholipid metabolite levels can aid in cancer diagnosis, prognosis, and treatment response monitoring.

**Figure 1:**
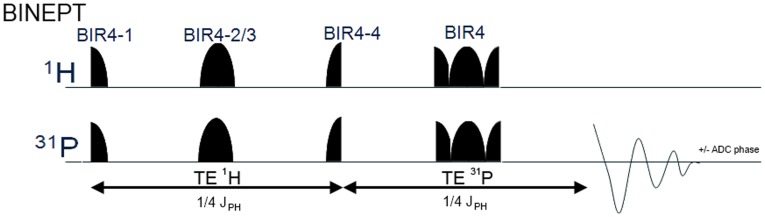
Schematic overview of the adiabatic version of the refocused insensitive nuclei enhanced by polarization transfer (BINEPT) sequence. Segmented BIR4 pulses (400 µs per segment, 35 ppm band width) and a full BIR4 180° pulse (400 µs, 35 ppm band width) were used instead of conventional 90 and 180 degree pulses. The echo time of 1/4 J was set to 34 ms, which gave optimal signals for the acquisition of PE and PC. Abbreviations: BIR4, B1 insensitive refocusing; J_PH_, J-coupling constant; TE^1^H, effective echo time for proton refocusing; TE^31^P, effective echo time for phosphorus refocusing.

Another way to improve the SNR, baseline flatness, and detection accuracy of PMEs and PDEs is to apply polarization transfer (PT) methods [11] such as refocused insensitive nuclei enhanced by polarization transfer (RINEPT) [12], [13]. PT techniques transfer the polarization of the excited ^1^H spins through J-coupling to the ^31^P spins during the echo time period TE^1^H as shown in Fig. 1, which increases the SNR and removes all resonances without ^1^H-^31^P coupling, thereby flattening the baseline. Previous studies at lower field strengths have focused on the ability of ^1^H-^31^P PT to increase the SNR. At 1.5T the SNR of PC obtained by using INEPT was comparable to that obtained by pulse-acquire (PA) acquisition [12]. However, for PDEs the SNR gain was 2 when using RINEPT [11]. At 3 T an SNR gain of 2.6, 1.6, 2.2, and 2.4 for PE, PC, GPE, and GPC, respectively, was reported when comparing a selective-RINEPT to PA acquisition in human brain [10]. At 7 T the concept of PT was expanded by combining it with a direct ^31^P detection sequence in one repetition time, which increases the SNR per unit of time as compared to a PT sequence alone [9].

As shown in the schematic overview of the adiabatic version of the refocused insensitive nuclei enhanced by polarization transfer (BINEPT) sequence in Fig. 1, the optimal duration for TE^1^H to achieve polarization transfer is relatively long due to the small J-coupling constants between ^31^P and ^1^H spins, which causes substantial signal loss to molecules that have short T2 values for transverse relaxation. Consequently, apart from a higher SNR for PE, PC, GPE, and GPC, PT will simultaneously decrease the SNR of larger ^31^P-containing molecules (with H-P coupling) that typically exhibit short T2 values and may overlap with these phospholipid metabolites [14]. This results in a flat baseline, which improves the detection of PMEs and PDEs.

To test these considerations, we performed PT at the high field strength of 9.4 T on a preclinical scanner. We demonstrate that an optimized PT technique at 9.4T results in a flat and clean baseline without loss of sensitivity compared to Ernst-angle pulse-acquire acquisition, and in an improved *in vivo* detection and quantification of PC, PE, GPE, and GPC in two breast cancer xenograft models.

## Materials and Methods

### Breast cancer xenograft models

Highly aggressive MDA-MB-231 and weakly aggressive MCF-7 breast cancer cells were purchased from the American Type Culture Collection and cultured as previously described [15]. 2×10^6^ MDA-MB-231 or MCF-7 cells in 50 µL of Hank's balanced salt solution (HBSS, Mediatech) were injected into the upper right thoracic mammary fat pad of anesthetized female athymic nude mice with an average weight of 26 g. Estrogen-dependent MCF-7 tumor growth was supported by a 0.18-mg 17β-estradiol 60-day release pellet (Innovative Research of America, Sarasota, FL) implanted subcutaneously into the back of mice 1 week before tumor inoculation. We studied 10 MCF-7 tumors of 1.67±0.59 cc that developed within nine weeks and 10 MDA-MB-231 tumors of 0.94±0.09 cc that developed within seven weeks. The maximum tumor size threshold for animals to be sacrificed was 2.5 cc. All surgical procedures and animal handling were approved by the Johns Hopkins University Institutional Animal Care and Use Committee, and conformed to the NIH Guide for the Care and Use of Laboratory Animals.

### Non-invasive in vivo ^31^P MRS studies

*In vivo* ^31^P MRS was performed on a 9.4T Bruker Horizontal Bore Small Animal Scanner (Bruker BioSpin Corp.). A ^1^H/^31^P double-tuned solenoid coil with an inner diameter of 12 mm was used (MRcoils BV, Drunen, The Netherlands). The tumor was hanging into the coil while the animal lay on a platform above the coil with an opening for the tumor [16]. Mice were anesthetized by breathing isoflurane (2% in air) through a nose cone. Body temperature was maintained by using a blanket with circulating warm water. Breathing rate was monitored with a movement sensor attached to the abdomen of each mouse. 3D RARE images were acquired using an echo time (TE) of 7.2 ms, repetition time (TR) of 500 ms, RARE factor of 4, flip angle of 90°, field of view (FOV) of 1 cmx1 cmx1 cm, 64 phase encode steps (64×64×64), and number of averages (NA) of 4. 1^st^ and 2^nd^ order B_0_ shimming was performed manually by minimizing the water line width of the entire tumor. Non-localized PA ^31^P MR spectra were acquired with adiabatic excitation (BIR4 45°, 200 µs, 120 ppm bandwidth), repetition time of 1 s, and 2000 averages, using a saturation slab to eliminate signals from adjacent muscles [17]. The combination of a short TR and 45° flip angle approximates PA acquisition with Ernst-angle excitation for PE and PC. Subsequently, an MR spectrum was acquired using an adiabatic version of the refocused insensitive nuclei enhanced polarization transfer technique BINEPT with a repetition time of 1 s and 2000 averages, which had an equal acquisition time of 33 min 20 s as PA. Segmented BIR4 pulses and a full BIR4 180° pulse were used as shown in Fig. 1. Both TE^1^H and TE^31^P were set to 34 ms (Fig. 1), which was the optimal echo time for the detection of PC and PE [18].

### Quantification of in vivo ^31^P MRS data

**Figure 2:**
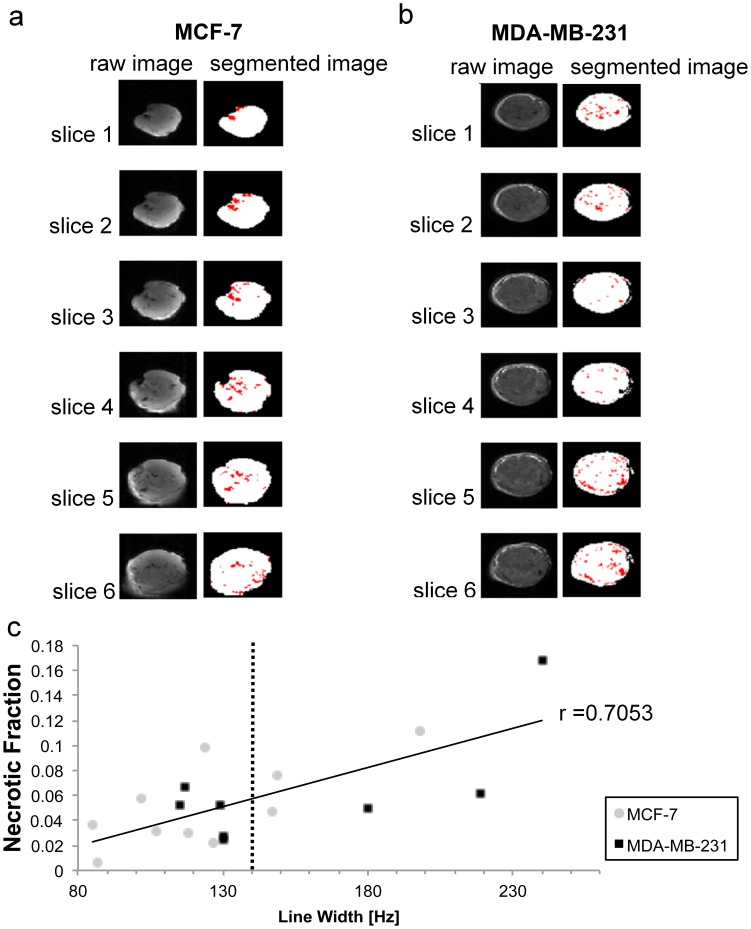
Raw RARE images and segmentation of corresponding slices. Necrotic tissue is depicted in red. Segmentation of (a) representative MCF-7 tumor and (b) representative MDA-MB-231 tumor. (c) Correlation between line width of PCr and necrotic fraction for all MCF-7 and MDA-MB-231 tumors measured.

Lorentzian lines were fitted to the PA and BINEPT ^31^P MR spectra using JMRUI 4.0 software [19] and the AMARES algorithm [20]. For PA spectra, the phosphocreatine (PCr) resonance was set to 0 ppm, and line widths of PE, PC, GPE, and GPC were constrained to that of PCr during fitting (Fig. 2). In the BINEPT spectra, which only contained PE, PC, GPE, and GPC, line width was constrained to that of PE, and the chemical shifts of PE and GPC were estimated by JMRUI using a starting value of 6.8 ppm for PE and 2.9 ppm for GPC (Fig. 2). Frequency differences between the resonance positions of PC and PE, and GPC and GPE were fixed to 100 Hz in PA and BINEPT spectra. To be able to compare MR spectra of different mice with different coil loads and gain settings, metabolite levels were quantified as ratios to the noise, which was measured from the standard deviation of the last 200 points in the time domain signal. PE, PC, GPE, and GPC levels from the PA spectra were corrected for differences in T1 relaxation. ^31^P T1 values of PE, PC, GPE, and GPC were measured *in vivo* by progressive saturation series in four MDA-MB-231 tumors as shown in Table 1. PE, PC, GPE, and GPC levels from the BINEPT spectra were corrected for polarization transfer efficiency (about 50%) and for ^1^H T1, ^1^H T2, and ^31^P T2 relaxation with estimates based on literature values. ^1^H relaxation times of tCho were based on values obtained at 7 and 9.4 T [21], [22], and ^31^P T2 relaxation times were based on values obtained at 3 and 7 T [10], [23], [24] (Table 1).

**Table 1:** *In vivo* ^31^P relaxation times.

		^31^P T1 relaxation time [s]				^31^P T2 relaxation time [ms]			
B0	Tissue	PE	PC	GPE	GPC	PE	PC	GPE	GPC
9.4 Tesla	MDA-MB-231 xenografts	3.4±0.9	2.1±1.0	2.2±0.8	NA	NA	NA	NA	NA
7 Tesla	Human breast *	4.7±1.2	5.6±0.8	NA	NA	192±46	201±85	84±82	102±138
7 Tesla	Human prostate **	7.6±1.5	5.9±1.3	8.3	5.9±1.7	NA	NA	NA	NA
3Tesla	Human brain ***	6.7±0.5	5.3±1.4	7.8±0.9	7.0±0.5	263±21	263±53	147±12	171±13

* T1 values from [29], T2 values from [23] and [24]

** Values from [44]

*** Values from

### Dual-phase extraction of tumors

Directly after *in vivo* MRS measurements, mice were deeply euthanized as verified by toe pinch, and sacrificed by cervical dislocation. The entire tumor was immediately removed, freeze-clamped, carefully weighed for quantification, pulverized in liquid N_2_, and homogenized in 4 mL of ice-cold methanol with a tissue tearor homogenizer. Both lipid and water-soluble tumor extract fractions were obtained using a dual-phase extraction method based on methanol/chloroform/water (1∶1∶1; v/v/v) as previously described [25], [26].

### ^31^P high-resolution (HR)-MRS studies

The lyophilized, water-soluble extract fractions were dissolved in D_2_O containing 0.24×10^−6^ mol 3-(trimethylsilyl)propionic-2,2,3,3-d4 acid (TSP; Sigma-Aldrich) and 6.0×10^−6^ mol phenylphosphonic acid (PPA; Sigma-Aldrich) as internal reference concentration and chemical shift standards. ^31^P HR-MRS was performed on a Bruker Avance 500 (11.7 T) spectrometer (Bruker BioSpin Corp.) using a 10-mm ^31^P probe. The MR spectra were acquired using the following acquisition parameters: 60° flip angle, 10162 Hz sweep width, 8 K time domain size, repetition time of 3 s and 2500 averages. The spectra were processed using the MestReC 4.9.9.6 software (Mestrelab Research). Lorentzian lines were fitted to the signals of PPA, PE, PC, GPE, and GPC. The areas under the curves were corrected for differences in T1 relaxation time and potential saturation effects. To this end, we measured the T1 relaxation times of PPA, PE, PC, and GPC with a progressive saturation series in a phantom solution in D_2_O. The T1-corrected PE, PC, GPE and GPC levels were normalized to tumor weight.

### MR data analysis

To assess the quality of our *in vivo* MR data, we measured the line width of PCr in the *in vivo* PA data of each tumor. In addition, we calculated the necrotic fraction of each tumor by using the dark areas in the corresponding 3D RARE T1-weighted images of each tumor [27], [28]. The tumor – air boundary was segmented in 3D using a threshold, set to the highest 10% in the histogram. Necrotic dark spots inside the tumor were segmented by manually adjusting a threshold to approximately the lowest 10% in the histogram. Necrotic regions were quantified as necrotic fraction per tumor by counting the number of necrotic voxels in the dark spots inside the tumor divided by the total number of voxels inside the tumor. The correlation between PCr line width and necrotic fraction was measured for each tumor as shown in Figure 2. As the fitting of MR spectra becomes unreliable in cases where the line width is broad, the line width of PCr in the *in vivo* MR spectra was used as a criterion to exclude tumors with poor spectral resolution and large necrotic regions from further analysis. Data from tumors in which the line width of PCr in the *in vivo* PA MR spectrum was larger than 140 Hz were excluded from further analysis (vertical line in Fig. 2c), although when comparing the group results based on the data from all tumors, the conclusions do not change. This cutoff value in line width was a tradeoff between including as much data as possible and excluding data with poor quality.

### Statistical analysis

A two-sided t-test assuming unequal variances between metabolite levels or ratios was performed to test for statistically significant differences between MDA-MB-231 and MCF-7 tumors, or PA and BINEPT methods.

## Results

Our initial quality check of MR data demonstrated a strong positive correlation (Pearson correlation, r = 0.7053, P = 0.0011) between necrotic fraction and spectral line width as shown in Figure 2. This could be explained by the fact that in tumors with larger necrotic fractions more tissue-tissue transitions between viable and necrotic areas were present which degraded the B_0_ homogeneity, which in turn led to larger spectral line widths (Fig. 2).

**Figure 3:**
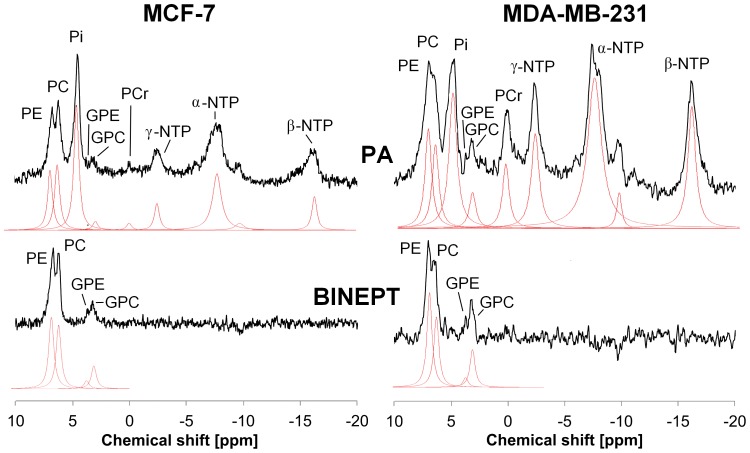
Example of *in vivo* pulse-acquire (PA, top) and BINEPT (bottom) ^31^P MR spectra of a representative MCF-7 (left) and MDA-MB-231 (right) tumor. Lorentzian lines as fitted by JMRUI are shown below each MR spectrum. All phosphorylated metabolites are visible in the PA spectrum, whereas the BINEPT spectrum only contains signals from phospholipid metabolites with H-P-coupling such as PE, PC, GPE, and GPC. Note the broad, uneven baseline in the 0–5 ppm region of the PA spectra, where signals from mobile membrane phospholipids are resonating [35], [45], [46]. The signal of β-NTP is formed by β-NTP only. The signal labeled α-NTP is an overlapping signal from α-NTP, α-NDP, NAD, and DPDE. The signal labeled γ-NTP is an overlapping signal from γ-NTP and β-NDP. Typically, β-NTP is the smallest peak of the three NTP signals, however, here, γ-NTP overlaps with a broad baseline signal that makes it appear smaller than β-NTP.

PA ^31^P MRS, as shown in Figure 3, detected the PMEs PE and PC and the PDEs GPE and GPC, in addition to inorganic phosphate (Pi), PCr, and α-, β-, and γ-nucleoside triphosphates (NTP). Data from mice that did not pass the initial spectral quality check (line width of PCr<140 Hz) were removed from further quantification, which resulted in n = 6 for MDA-MB-231 and n = 7 for MCF-7 tumors. BINEPT ^31^P MRS only detected PE, PC, GPE, and GPC. The PDE signals of GPE and GPC were better visible in the BINEPT spectra compared to the corresponding PA spectra as no overlapping signals from sugar phosphates, large membrane phospholipids, and other phosphorylated compounds were detected in the 0–10 ppm region, leading to a flat baseline throughout the spectrum (Fig. 2). Also, the PME in BINEPT spectra were not overlapping with other phospholipid signals.

**Figure 4:**
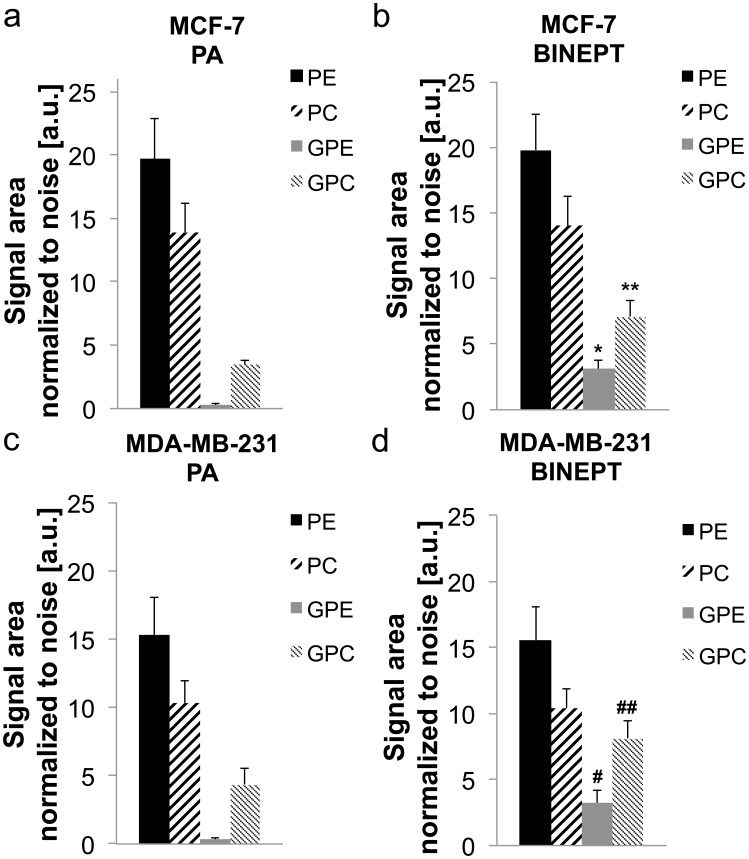
*In vivo* phospholipid metabolite levels as measured by (a, c) PA and (b, d) BINEPT ^31^P MRS. Average metabolite levels ± standard error are shown for (a, b) MCF-7 (n = 7) and (c, d) MDA-MB-231 (n = 6) breast tumor xenografts. Statistical results: PA vs BINEPT MCF-7: GPE p = 0.004 *, GPC p = 0.03 **; PA vs BINEPT MDA-MB-231: GPE p = 0.02 ^#^, GPC p = 0.06 ^##^; MCF-7 vs MDA-MB-231: PA, for all metabolites p>0.20; MCF-7 vs MDA-MB-231: BINEPT, for all metabolites p>0.21.

Figure 4 shows the quantification of the *in vivo* levels (average ± standard error) of PE, PC, GPE, and GPC obtained with PA and BINEPT ^31^P MRS. We did not detect any significant differences in PE, PC, GPE, or GPC levels in MDA-MB-231 (n = 6) *versus* MCF-7 (n = 7) tumors. However, BINEPT ^31^P MRS detected higher levels of GPE and GPC as compared to the detection by PA ^31^P MRS within the same tumor model. This was the case for both MBA-MB-231 as well as MCF-7 tumors. The pH of these tumors as determined from the chemical shift difference between PCr and Pi was 6.92±0.10 for MCF-7 tumors and 7.00±0.11 for MDA-MB-231 tumors, which was not significantly different from each other.

**Figure 5:**
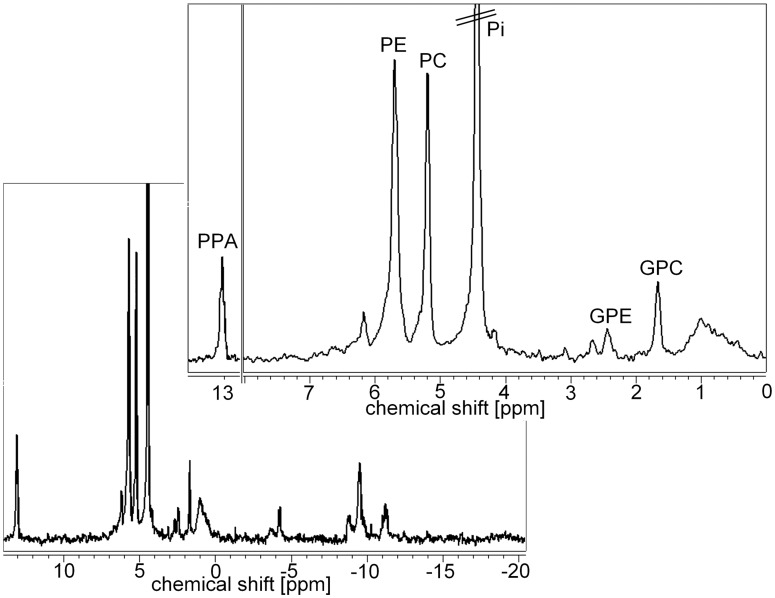
^31^P High-Resolution MR spectrum of the same MCF-7 tumor as shown in Fig. 2. The full spectral range is displayed in the spectrum on the bottom. Signals of NTPs are visible in the 0 to -15 ppm region. An expanded region of 0 to 8 ppm is displayed on the top, as well as the region with the PPA reference signal at 13 ppm. The signals of PE, PC, GPE, and GPC are clearly separated.

**Figure 6:**
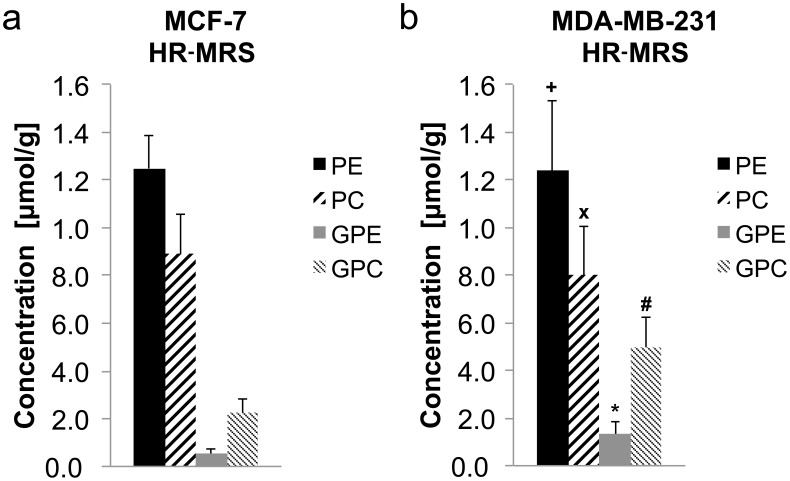
Quantification of PE, PC, GPE, and GPC levels from ^31^P HR-MRS of (a) MCF-7 and (b) MDA-MB-231 breast tumor extracts. Average metabolite levels ± standard errors are shown and were expressed as concentration in μmol per gram of tumor tissue. Statistical results: MCF-7 vs MDA-MB-231: PE p =  0.22 ^+^, PC p = 0.77 ^x^, GPE p = 0.31 *, GPC p = 0.16 ^#^.

Figure 5 shows a representative ^31^P HR-MR spectrum of the aqueous phase of an MCF-7 breast tumor extract. The PME signals PE and PC as well as the PDE signals GPE and GPC were clearly separated in the ^31^P HR-MR spectra of tumor extracts (Fig. 5). Figure 6 shows the quantification of the PE, PC, GPE, and GPC concentration in MCF-7 and MDA-MB-231 tumors obtained from the ^31^P HR-MR spectra. When normalized to tumor weight and the concentration reference PPA, no statistically significant differences in PE, PC, GPE, and GPC concentrations were detected between MBA-MB-231 (n = 6) and MCF-7 (n = 7) tumors (Fig. 6).

**Figure 7:**
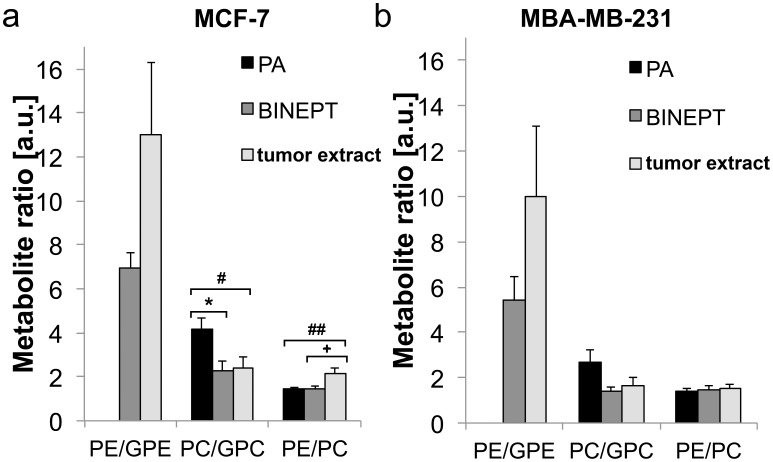
Comparison of phospholipid metabolite ratios between *in vivo* PA, *in vivo* BINEPT, and tumor extract HR ^31^P MRS measurements of the same (a) MCF-7 and (b) MDA-MB-231 tumors. Values are given as average ± standard error. The PE/GPE ratios are not shown for *in vivo* PA measurements because the GPE resonance could not be reliably detected in several PA spectra due to low SNR. Statistical results: PA vs BINEPT: MCF-7 PC/GPC p = 0.02 *, all other ratios in MCF-7 and MDA-MB-231 p>0.12; PA vs tumor extract: MCF-7 PE/PC p = 0.04 ^#^, PC/GPC p = 0.05 ^##^, all other ratios in MCF-7 and MDA-MB-231 p>0.21; BINEPT vs tumor extract: MCF-7 PE/PC p = 0.05 ^+^, all other ratios in MCF-7 and MDA-MB-231 p>0.24.

Figure 7 shows a direct comparison between the *in vivo* PA, *in vivo* BINEPT, and tumor extract ^31^P HR-MRS measurements, which were taken sequentially from the same tumors. The PE/GPE ratio could not be assessed accurately in the PA spectra because GPE was barely detectable, resulting in very small GPE values, leading to high ratios of PE/GPE and large standard deviations, particularly in MDA-MB-231 tumors. There was a difference between PC/GPC ratios obtained from *in vivo* BINEPT spectra and PA spectra. The value from BINEPT spectra more closely resembled the PC/GPC ratio that was obtained by HR-MRS of tumor extracts, for MCF-7 as well as MDA-MB-231 tumors. The PE/PC ratios obtained from *in vivo* PA and BINEPT measurements were similar. No significant differences were detected between any of the phospholipid metabolite ratios of MCF-7 *versus* MDA-MB-231 tumors. However there was a trend towards a higher PE/PC ratio in MCF-7 tumors as compared to MDA-MB-231 tumors.

## Discussion

The polarization transfer technique BINEPT significantly improved the *in vivo* detection of the phospholipid metabolites PE, PC, GPE and GPC at high magnetic field strength in human breast tumor xenografts. The BINEPT sequence removed all signals originating from ^31^P compounds without P-H coupling because its detection is based on P-H polarization transfer. As a consequence, PCr, Pi, and NTP signals, as well as signals from sugar phosphates and large membrane phospholipids with broad line widths in the spectral region between 0 ppm and 10 ppm, were not detected by BINEPT, leading to a flat baseline, which significantly improved the quantification of PE, PC, GPE and GPC by enabling the use of simple line-fitting algorithms.

An alternative way to obtain a flat baseline could be the use of an off resonance saturation pulse. The effect of such a pulse would be that magnetization transfer and chemical exchange would eliminate broad resonances from very large molecules [7]. However, the signal intensities of PE, PC, GPE, and GPC would be attenuated by such a pulse due to magnetization transfer, whereas BINEPT enhances these signal intensities. Proton decoupling applied during acquisition could improve the spectral resolution and SNR. However, at 9.4 Tesla the chemical shift anisotropy is the dominant relaxation mechanism, which will only result in a marginal increase of SNR, as the water line width is in the order of 80 Hz and the J-coupling in PMEs and PDEs is ∼7 Hz. In addition, the possible signal enhancement by nuclear overhauser enhancement (NOE) effect is different for each metabolite and spatially heterogeneous due to the use of a surface coil. This will introduce more variation into the data, which is not desired, and therefore proton decoupling and NOE were not applied in this study. Although the polarization transfer efficiency can also be different between compounds, this efficiency is easily calculated using spin operator calculations, and can therefore be taken into consideration during quantification. Only differences in T2 relaxation will attribute to an unknown variance in polarization transfer efficiency. However, recent innovations in pulse sequence design may even recover this missing information in T2 [23].

Considering absolute quantification, it is important to note that BINEPT, unlike PA, does not depend on the ^31^P T1 relaxation, but rather on the T1 relaxation of ^1^H and the T2 relaxation of ^31^P and ^1^H spins. We measured the ^31^P T1 relaxation times at 9.4 T *in vivo* and estimated the ^31^P T2 relaxation time to be 150 ms for PE and PC and 80 ms for GPE and GPC based on values reported in the brain at 3 T and the breast at 7 T [24], [29] (Table 1). The ^1^H T1 relaxation time of the tCho resonance in the brain at 9.4 T is approximately 1400 ms [21] and its ^1^H T2 relaxation time at 7 T has been reported to be between 150 and 200 ms [22], [30]. Therefore, we assumed the ^1^H T1 relaxation time of PE, PC, GPE and GPC in the tumor at 9.4 T to be 1400 ms, we estimated their T2 relaxation time to be 120 ms (slightly lower than at 7 T). The fact that we used such estimates made it questionable to directly compare the quantified phospholipid metabolite levels from *in vivo* BINEPT, *in vivo* PA, and tumor extract HR ^31^P MRS measurements.

BINEPT ^31^P MRS detected higher levels of GPE and GPC as compared to the detection by PA ^31^P MRS within the same tumor model. This underestimation of GPE and GPC levels by PA is most likely due to limitations of fitting with the JMRUI algorithm, which typically underestimates the fitting of signals with narrow line width and low SNR, such as GPE and GPC, which are overlapping with other broad signals. The ^1^H and ^31^P T2 relaxation times used in the quantification are influencing the final metabolite levels measured from the BINEPT spectra displayed in Figure 4. The relaxation times of GPE and GPC are typically less accurately measured than the relaxation times of PE and PC, which have a much higher concentration in most tissues and hence appear with higher SNR. Therefore, the PE/GPE and PC/GPC ratios were measured more reliably by BINEPT than by PA ^31^P MRS *in vivo*. BINEPT ^31^P MRS was best suited for the detection of the PE/GPE ratio since in contrast to the spectra obtained with PA, both PE and GPE appeared with sufficient SNR in the BINEPT spectrum. Accurate detection of PE/GPE and PC/GPC ratios is of great importance in studying cancer tissues, as these ratios are strongly associated with cancer aggressiveness [2], [6].

Comparing ratios of metabolites in tumor extracts and *in vivo* measurements showed that the PC/GPC ratio obtained with BINEPT better resembled the tumor extract PC/GPC ratio than did the PC/GPC ratio obtained with PA. The quantification of BINEPT MR spectra using estimated values for relaxation times could be used in longitudinal studies on the effects of a given anticancer treatment on choline and ethanolamine metabolism.

The PC/GPC ratios detected in this study are in good agreement with a recent study of Morse *et al* [31], in which MCF-7 tumors also displayed a slightly higher PC/GPC ratio compared to that of MDA-MB-231 tumors. These findings do not match the PC/GPC levels reported in the MCF-7 and MBA-MB-231 breast cancer cell lines grown in cell culture [6]. This is not surprising in light of the various differences that exist between cell lines in culture *versus* tumor xenografts. The PE/PC ratios in MCF-7 and MDA-MB-231 breast tumor xenografts in our study were comparable at around 1.5, which points towards a similar activation in choline and ethanolamine phospholipid metabolism in both tumor models, with a higher level of PE than PC. This is in good agreement with the finding that choline kinase alpha, which is overexpressed and activated in human breast cancers [32], has the ability to use ethanolamine as well as choline as substrates to produce PE and PC, respectively [33].

In early attempts with *in vivo* ^31^P MRS of human cancers, a combined peak for PME and PDE was detected [34], [35]. Some early studies demonstrated the potential of ^31^P MRS for monitoring the effects of anticancer treatments [36], [37] by evaluating the ratio of PME to PDE, and for assessing proliferation by evaluating the ratio of PME to γ-NTP [38]. However, at that time, the spectral resolution was insufficient for resolving the individual metabolites of PE, PC, GPE and GPC. With recent technological developments of improved human high-field MR scanners, coils, and pulse sequences for ^31^P MRS, resolving these individual metabolites has become feasible in the clinical setting [9], [13]. Furthermore, using human tissue biopsies and magic angle spinning ^31^P HR-MRS, it has been possible to use phospholipid metabolites as markers to discriminate between tumor types and grades [39], [40], evaluate response to treatment [36], predict response to treatment [41], and assess resection margins during breast cancer surgery [42].

As ^1^H MRS is more sensitive than ^31^P MRS, it has been more widely used to study tCho in breast cancer. tCho has been used as a marker for malignancy and treatment response, however, the data from different groups show large variability and discrepancy between the findings [3]. Moreover, the ^1^H MRS detection of tCho in shrinking tumors often fails due to large signals from lipids [43] and poor magnetic field homogeneity (B_0_), both of which are hampering accurate detection of tCho levels in breast lesions. This inaccuracy in tCho detection is particularly limiting for the assessment of tCho endpoints in longitudinal studies of response to treatment [3]. As ^31^P MRS does not pose the problem of large overlapping lipid signals and provides detailed information on anabolic and catabolic metabolites in phospholipid metabolism such as PE, PC, GPE, and GPC, it may be a better-suited technique to evaluate the role of choline- as well as ethanolamine-related metabolites in breast cancer. Even though the sensitivity of ^31^P MRS is relatively low, the PME levels in tumors can be very high, which allows for the possibility of assessing phospholipid metabolism even in small tumors. Furthermore, in the near future, better coils and improved pulse sequences will become available for clinical use, which will increase the sensitivity of ^31^P MRS. Finally, the ability to detect the individual phospholipid metabolites PE, PC, GPE, and GPC would significantly improve the diagnosis, treatment monitoring, and assessment of tumor response to therapy of breast tumors.

## Supporting Information

Checklist S1 **ARRIVE Checklist.**
(DOC)
